# Mitigation of Copper Stress in Maize by Inoculation with *Paenibacillus polymyxa* and *Bacillus circulans*

**DOI:** 10.3390/plants9111513

**Published:** 2020-11-08

**Authors:** Arafat Abdel Hamed Abdel Latef, Abbu Zaid, Abo-Baker Abd-Elmoniem Abo-Baker, Wesam Salem, Mona Fawzy Abu Alhmad

**Affiliations:** 1 Biology Department, Turabah University College, Turabah Branch, Taif University, P.O. Box 11099, Taif 21944, Saudi Arabia; 2 Botany and Microbiology Department, Faculty of Science, South Valley University, Qena 83523, Egypt; wesam.salem@svu.edu.eg (W.S.); mfmahmoud@tu.edu.sa (M.F.A.A.); 3 Plant Physiology and Biochemistry Section, Department of Botany, Aligarh Muslim University, Aligarh 202002, India; azaid@myamu.ac.in; 4 Department of Soils and Water Science, Faculty of Agriculture, South Valley University, Qena 83523, Egypt; Bashaabobaker@yahoo.com; 5 Biology Department, Faculty of Science, Taif University, Al-Hawiyah, Taif 21944, Saudi Arabia

**Keywords:** antioxidant activities, *Bacillus circulans*, copper toxicity, maize, *Paenibacillus polymyxa*, plant growth-promoting rhizobacteria (PGPR)

## Abstract

Copper (Cu) is a micronutrient that assumes a principal role in plant growth and development. However, its excess concentration in soil is imperiling crop productivity. Inoculation with different bacterial strains in cereals could modify growth traits, photosynthetic effectiveness, and generation of strong antioxidant defense systems to make them more tolerant of Cu stress. Therefore, a pot study was designed to test plant growth-promoting rhizobacteria (PGPR) including *Paenibacillus polymyxa* and *Bacillus circulans* to Cu exposed maize (*Zea mays* L.) plants. Increasing Cu (100 to 500 µM of CuSO_4_) concentration decreased growth traits, photosynthetic pigments, soluble sugars, phosphorous (P) and potassium (K) contents, and the activity of catalase (CAT) but increased proline and malondialdehyde (MDA) content, the activity of peroxidase (POD) and Cu ions at root and shoot level. Moreover, the bacterial treatment also modulated the antioxidant capability in stress-free plants. Nevertheless, inoculation with *P. polymyxa* and *B. circulans* alleviated Cu-induced growth, photosynthetic pigments and mineral nutrient (P and K) on one hand and regulating the pools of osmolytes and antioxidant enzymes, whilst simultaneously reducing MDA and Cu root and shoot contents. These improved activities of antioxidant enzymes and the regulation of osmolytes content elicited by the blend of bacterial inoculation would have retained the ability of maize plants to confer resilience to Cu stress. This study further affirms that the application of two specific bacterial strains to maize plants proved very effective to ameliorate the Cu toxicity.

## 1. Introduction

Due to changing environmental conditions, the problem of heavy metal (HM) stress in the plant–soil environment has resulted in a considerable reduction of growth and development of major crop plants worldwide [1,2,3,4,5]. Furthermore, pollution caused by HMs and improper fertilization practices poses a serious threat to a sustainable environment and healthy food. Copper is one of eight essential microelements and is an important part of the cofactor of various metalloproteins and other macromolecules involved in wide essential metabolic processes in plants including net photosynthesis, respiration, lignification of the cell wall, and various protective mechanisms [6,7,8]. However, owing to various anthropogenic activities, this HM has gained particular attention among plant stress physiologists due to its dual nature in the plant system: essential as well as toxic at an optimum and high level respectively [9,10]. An excess of Cu concentration in plant tissues induces oxidative stress via enhanced biosynthesis of various reactive oxygen species (ROS), resulting in damage to photosystem pigment proteins, DNA, RNA, lipids, enzymes, altered thylakoid membranes composition, decreased chlorophyll and mineral nutrient contents and hampered meristems development [11,12,13,14,15]. Hence, given its dual nature (essential as well as toxic), this HM could involve complex mechanisms of uptake, transport, sequestration and detoxification inside the plant tissues at the cellular level. However, several efforts have been made to counteract the Cu-induced toxicity in diverse crop plants.

In recent times, as a result of the rising costs of chemical fertilizers as well as the related environmental risk and health issues associated with the use of such compounds, a severe degradation in soil fertility and a decrease in the quality of foods and their products have been observed. Therefore, the researchers’ mentality has shifted to the use of biological solutions, such as microbial biotechnology to solve the HM-toxicity problems [16,17,18]. Moreover, there is a growing desire to understand modifications in microbial rhizosphere diversity and community structure under different environmental conditions. The rhizospheric microbes are typically classified into three types: useful, harmful, and inert [19] and play an important role in mitigating diverse plant biotic and abiotic pressures [20,21]. Plant growth-promoting rhizobacteria (PGPR) are agriculturally important in increasing plant growth and yield [22,23]. Several PGPRs have been evidenced to alleviate the toxicity of HM in crop plants. In addition, inoculation technology involving PGPR is recognized as a beneficial eco-friendly tool to optimize sustainability in agriculture and allied sectors due to its low costs and biological nature compared with other industrial inputs [17]. It has been reported that inoculation with two *Bacillus* isolates enhanced tolerance to salt, drought, and HM stresses in potato plants [24]. In soybean plants, *Bacillus cereus*, *Bacillus megaterium*, *Trichoderma longibrachiatum* and *Trichoderma simmonsii* boosted tolerance to simultaneous salt and drought stresses [25]. *Staphylococcus arlettae* strain *MT4* has been shown to alleviate Cr toxicity in sunflower plants by restricting its uptake and strengthening the plant antioxidant defense system [26]. In pennyroyal, *Azotobacter* and *Azospirillum* strains increased the biosynthesis of secondary metabolites and imparted drought stress tolerance [27]. Moreover, the strain of *Bacillus* was found to diminish the stress induced by nanoparticles in mustard plants [28]. In alfalfa plants, salt tolerant PGPR enhanced salt stress tolerance under high salinity conditions [29]. In earlier reports, it has been concluded that maize seedlings inoculated with PGPR have shown tolerance to salt [30,31], Al and salt [32], Cd [33], drought [34] and chilling [35] stresses. Application of *Bacillus siamensis* to wheat plants showed improved tolerance to Cd stress by restricting the accumulation of Cd and boosting the antioxidant defense system [36]. However, research regarding underlying mechanisms of inoculation of maize plants with phosphate- and potassium-solubilizing bacterial strains *P. polymyxa* and *B. circulans* under Cu toxicity receive little attention.

Maize (*Zea mays* L.) is an important cereal crop cultivated worldwide and exhibits strong potential for the phytoremediation of HM contaminated soils [37,38]. Earlier studies also reported the toxic impact of Cu on maize plants [39,40,41,42,43]. However, more detailed studies should be done to obtain a precise nature of PGPR mediated Cu stress tolerance in maize plants involving various biochemical and physiological abilities. With this aim, the present study was undertaken.

## 2. Materials and Methods

### 2.1. Bacterial Inoculation and Experimental Design

Two phosphate- and potassium-solubilizing bacterial strains *Paenibacillus polymyxa* and *Bacillus circulans* were utilized in the present study. *P. polymyxa* was locally isolated [44] whereas *B. circulans* was obtained from Microbial Resource Centre, Faculty of Agriculture, Ain Shams University, Egypt. Maize seeds (*Zea mays* L. cv. single hybrid 10) were first treated in NaOCl (10%, v/v) for 4 min followed by repeated washings with double distilled water (DDW) for five times. The bacteria (6 × 10^6^ cfu/gm) were inoculated at the rate of 10% of the weight of maize seed and mixed thoroughly until the seeds were found to be uniformly surface coated with bacterial strains [45,46,47,48]. After inoculation, the sterilized maize seeds were sown in plastic pots (5 seeds per pot). The pots contained 2 kg of an autoclaved dry clay soil. There were three replicates per treatment (*n* = 3) and the plastic pots were arranged into completely randomized block design in a factorial arrangement. In total there were nine treatments: (i) Control, plants without copper stress and bacterial strains inoculation; (ii) Cu 1: plants exposed to 100 µM CuSO_4_; (iii) Cu 2: plants exposed to 500 µM CuSO_4_; (iv) *P. polymyxa* (*Pp*) inoculated plants without CuSO_4_; (v) Cu 1 + *Pp*: plants exposed to 100 µM CuSO_4_ and inoculated with *P. polymyxa*; (vi) Cu2 + *Pp*: plants exposed to 500 µM CuSO_4_ and inoculated with *P. polymyxa*; (vii) *B. circulans* (*Bc*) inoculated plants without CuSO_4_; (viii) Cu 1 + *Bc*: plants exposed to 100 µM CuSO_4_ and inoculated with *B. circulans*; (ix) Cu 2 + *Bc*: plants exposed to 500 µM CuSO_4_ and inoculated with *B. circulans*. Copper in the form of copper sulfate (CuSO_4_.5H_2_O, 99% purity, Sigma-Aldrich, St. Louis, MO, USA) was given as the point of supply of Cu stress through the soil at different doses of 100 or 500 µM at 10 days after sowing (DAS). From the first DAS, the pots of all the treatments were given 400 mL DDW as and when required. The experimental pots were placed under field conditions with a day length of 10–12 h, mean temperatures of 35/22 °C, and relative humidity of 55–67% in the wire-house of the experimental farm of the South Valley University, Qena, Egypt. After 25 days of seed germination, maize plants were harvested for examining different growth and physio–biochemical traits as follows.

### 2.2. Growth Parameters

After harvesting, the fresh weight (FW) of maize plants was measured. The maize seedlings were then oven-dried at a constant weight and the dry weight (DW) was estimated. The leaf area (LA) of maize seedlings was estimated using a planimeter (SOKKIA Planimeter KP-90, London, UK).

### 2.3. Photosynthetic Pigment Analysis

The photosynthetic pigments viz-chlorophyll (Chl a and b) and carotenoids (Caro) were determined by adopting Lichtenthaler and Wellburn [49] protocol. Fresh leaf material (100 mg) was pelleted in a pre-chilled mortar with 4 mL of acetone solution (80%, v/v). The slurry was centrifuged at 3000 rpm for 5 min. The pellet was discarded and the absorbances of resulted supernatants were measured spectrophotometrically at 663, 645, and 470 nm for determining the Chl a, b and Caro respectively against the acetone (80%, v/v) taken as blank.

### 2.4. Osmolyte Contents

The protocol of Irigoyen et al. [50] was adopted to estimate soluble sugars content. The content of soluble proteins was determined by following the Bradford [51] method. For the determination of proline, the protocol of Bates et al. [52] was followed. 

### 2.5. Determination of Malondialdehyde (MDA)

For MDA content determination, the thiobarbituric acid (TBA) reaction method described by Zhang and Qu, [53] was adopted. The complete experimental guide has already been given by Abdel Latef and Tran [54].

### 2.6. Antioxidant Enzymes Activity

The freshly collected leaf samples were harvested for the determination of various antioxidant enzyme activities including catalase (CAT; EC 1.11.1.6), peroxidase (POD; EC 1.11.1.7) and ascorbate peroxidase (APX; EC 1.11.1.11). The leaf tissue was taken in the pre-chilled mortar with liquid N_2_. After that, the enzymatic extraction was performed according to the method of Ahmad et al. [55]. The CAT activity was determined as per the protocol of Aebi [56]. The procedure proposed by Maehly and Chance [57] was used for the POD activity determination. The method of Chen and Asada [58] was utilized for the determination of APX activity. The detailed antioxidant enzymes’ procedure is described in Abdel Latef et al. [48].

### 2.7. Estimation of Phosphorous (P), Potassium (K) and Copper Contents

The dried seedling samples were acid digested for 12 h in 80% perchloric acid and concentrated H_2_SO_4_ solutions (1:5) at 140–160 °C. After that, the samples were cooled and diluted with 1 M HCl. Blanks were prepared without the samples. The procedure for determining K content was adopted by Williams and Twine [59] and the content of P was calculated using ammonium molybdate blue based on Allen [60] procedure.

The root and shoot samples were dried at 85 °C for 24 h, digested in a tri-acid (H_2_SO_4_, HNO_3_, HCLO_4_) mixture at the ratio of 5:1:1 v/v. Drops of HNO_3_ and H_2_O_2_ were added to make the solution transparent. The Cu content of root and shoot was determined by atomic absorption spectrophotometer.

### 2.8. Statistical Analysis

Data were analyzed by the analysis of variance (ANOVA) with SAS software (Version 9.1; SAS Institute, Cary, NC, USA), and Duncan’s multiple range test was calculated at the 0.05 level of significance (*p* ˂ 0.05). Data shown in the figures are the mean ± standard deviation (SD) of three independent replicates. Principal component analysis (PCA) was done by Minitab software by employing Minitab Release 19.2.0 statistical software.

## 3. Results

### 3.1. Inoculation with Bacteria Improves Growth Traits under Cu Stress

The weights (fresh and dry) of maize seedlings were decreased under both Cu (100 and 500 µM) doses versus control. The inoculation with *Pp* and *Bc* increased the FW and DW under stress-free conditions compared to controls. Inoculation with *Pp* and *Bc* provoked a significant increase in plants receiving Cu 1 and 2 doses in FW and DW compared to Cu 1 and 2 plants respectively (Table 1).

A negative effect in the case of the LA of maize seedlings was noticed under Cu 1 and 2 conditions compared to control plants. The plants inoculated with *Pp* and *Bc* caused a marked increase in LA and the maximum increase was noticed under stress-free conditions with *Pp* compared to control plants. The *Bc* and *Pp* inoculated plants grown under both Cu doses also recorded a significant increase in LA over Cu 1 and 2 plants (Table 1).

### 3.2. Bacterial Inoculation under Cu Doses Increases the Contents of Chlorophyll a, b and Carotenoids

The content of Chl *a* was decreased under both Cu 1 and Cu 2 concentrations against controls but this decrease was more pronounced in the Cu 2 dose. The *Pp* and *Bc* inoculation increased the Chl *a* content under non-stress conditions relative to control plants. In the case of Cu 1 conditions, the inoculation with *Pp* and *Bc* maximally improved the Chl *a* in comparison to Cu 1 alone plants, whereas, a smaller increase was noticed in Cu 2 plants inoculated with *Pp* and *Bc*, respectively compared to Cu 2 alone plants (Table 2).

In contrast to the contents of Chl *a*, the Chl *b* was found to be decreased significantly by Cu 2 concentration versus control and the effect was found to be non-significant at the Cu 1 dose. The inoculation with *Pp* improved the Chl *b* content and its effect was followed by *Bc* under non-stress conditions compared to control plants. In Cu 1 grown plants, the inoculation with *Pp* and *Bc* boosted the Chl *b* in comparison to Cu 1 alone plants. Cu 2 plants inoculated with *Pp* and *Bc* respectively also showed a significant increase in Chl *b* content compared to Cu 2 alone plants (Table 2).

Caro content remained non-significant up to Cu 1 then a significant decrease was observed in Cu 2 compared to the control. Plants inoculated with *Pp* and *Bc* registered a non-significant increase in Caro content compared to control plants. It was also found that Cu 1 + *Pp* and Cu 1 + *Bc* plants showed a non-significant increase in Caro content against Cu 1 alone plants. However, a significant increase was noticed in Cu 2 + *Pp* treatment compared to Cu 2 treatment alone (Table 2).

### 3.3. Bacterial Inoculation under Graded Levels of Cu Regulates Organic Solutes

The Cu 1 and 2 treatments resulted in a significant decrease in soluble sugars content compared to controls. The inoculation with *Pp* and *Bc* increased the soluble sugars content under non-stress conditions compared to control plants. Its effect was followed by Cu 1 + *Pp* compared to Cu 1 alone plants. A significant increase was also noted in Cu 2 + *Pp* and Cu + *Bc* inoculated plants compared to Cu 2 alone plants (Table 2).

The soluble proteins content was diminished under Cu 1, then a non-significant increase in soluble proteins content was noted in Cu 2 plants compared to controls. The inoculation with *Pp* and *Bc* resulted in a non-significant increase in soluble proteins compared to control plants under non-stress conditions. The maize plants grown under Cu 1 + *Pp* and Cu 2 + *Bc* showed a significant increase in the soluble proteins content compared to Cu 1 alone plants. The inoculation with *Pp* and *Bc* in Cu 2 registered a non-significant increase in soluble proteins content compared to Cu 2 treatment alone (Table 2).

A significant increase was noticed in the proline content of maize seedlings grown under Cu 1 and Cu 2 applications compared to controls. In contrast to soluble sugars and proteins, the *Pp* and *Bc* imposed a decreasing trend in proline compared to the control. The proline content showed a marked decreasing trend with *Pp* inoculation in Cu 1 and Cu 2 plants, respectively, in comparison to Cu 1 and Cu 2 alone plants. Our results also showed that a significant decrease in proline content was also recorded when Cu 1 and 2 plants were inoculated with *Bc* compared to Cu 1 and Cu 2 plants alone (Table 2).

### 3.4. Bacterial Inoculation Diminishes MDA Content under Cu Stress

Exposure of maize plants to Cu 1 and 2 doses showed a significant increase in MDA content and this accumulation was maximum in Cu 2 plants over control plants. Inoculation with *Pp* and *Bc* decreased the MDA under non-stress conditions versus the control. The *Pp* and *Bc* inoculation in Cu 1 and Cu 2 plants caused a dramatic decrease in MDA content compared to Cu 1 and 2 alone plants, respectively (Table 2).

### 3.5. Bacterial Inoculation Decreases Root and Shoot Cu Content in Maize Plants under Cu Stress

**Figure 1:**
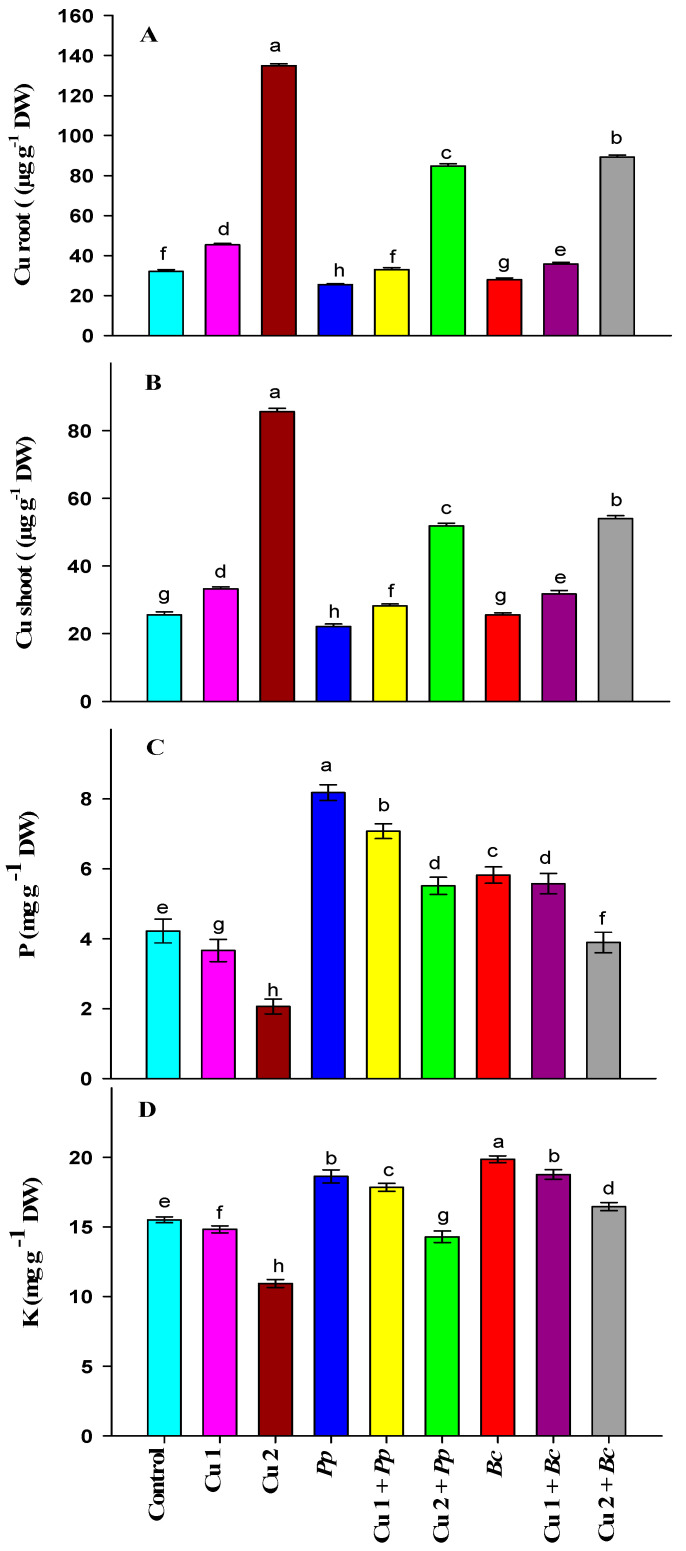
Effects of bacterial inoculation under copper stress on (**A**) copper in root (**B**) copper in shoot (**C**) phosphorous (P) and (**D**) potassium (K) in maize plants. Bars represent the standard deviation (±SD) of the means (*n* = 3). Different letters indicate significant differences among the treatments at *p* ˂ 0.05, according to Duncan’s multiple range test. The treatments include Control, plants without copper stress and bacterial strains inoculation; Cu 1: plants exposed to 100 µM CuSO_4_; Cu 2: plants exposed to 500 µM CuSO_4_; *P. polymyxa* (*Pp*) inoculated plants without CuSO_4_; Cu 1 + *Pp*: plants exposed to 100 µM CuSO_4_ and inoculated with *P. polymyxa*; Cu 2 + *Pp*: plants exposed to 500 µM CuSO_4_ and inoculated with *P. polymyxa*; *B. circulans* (*Bc*) inoculated plants without CuSO_4_; Cu 1 + *Bc*: plants exposed to 100 µM CuSO_4_ and inoculated with *B. circulans* and Cu 2 + *Bc*: plants exposed to 500 µM CuSO_4_ and inoculated with *B. circulans*.

Maize plants grown under Cu 1 and Cu 2 showed a significant increase in root Cu content over control plants. Inoculation with *Pp* and *Bc* decreased the root Cu content compared to control plants in stress-free plants. The inoculation of plants with *Pp* and *Bc* in Cu 1 and Cu 2 induced a significant decrease in Cu content compared to Cu 1 and 2 alone plants (Figure 1A).

In line with Cu in root, shoot Cu content registered a significant increase under Cu 1 and Cu 2 compared to the control plants. The bacterial inoculation with *Pp* caused a significant decrease in the shoot Cu content but a non-significant increase was noticed in plants inoculated with *Bc* versus the control plants. The Cu content in shoot decreased significantly in plants inoculated with *Pp* and receiving Cu 1 and Cu 2 respectively versus Cu 1 and Cu 2 alone treatments. Moreover, the *Bc* inoculation treatment in Cu 1 and Cu 2 plants also registered a significant decrease in shoot Cu content compared to Cu 1 and Cu 2 alone plants (Figure 1B).

### 3.6. Bacterial Inoculation under Cu Stress Increases P and K Contents

A significant decrement in the P content was noticed under both Cu 1 and Cu 2 doses compared to the control. The inoculation with *Pp* and *Bc* caused a significant increment in P versus the control treatment. A significant increase in P content was observed in *Pp* + Cu 1 and *Pp* + Cu 2 treatments compared to the respective Cu treatments. The treatments: *Bc* + Cu 1 and *Bc* + Cu 2 registered a significant increase in P content compared to Cu 1 and Cu 2 treatments (Figure 1C).

In line with P, K content also exhibited a significant decrease under Cu 1 and Cu 2 doses compared to the control. The Cu 2 then Cu 1 grown plants showed a maximum and more significant decrease versus control plants. The bacterial inoculation with *Pp* and *Bc* increased the K content over the control. In this case, the K content was found to be highest in plants inoculated with *Bc*. In Cu 1 and Cu 2 plants, inoculation with *Pp*, respectively, increased K content versus Cu 1 and Cu 2 alone treatments. The inoculation treatments with *Bc* in Cu 1 and Cu 2 plants also registered a significant increase in K content compared to Cu 1 and Cu 2 alone treatments (Figure 1D).

### 3.7. Bacterial Inoculation Modulates the Antioxidant Enzymes of Maize Plants under Cu Stress

**Figure 2:**
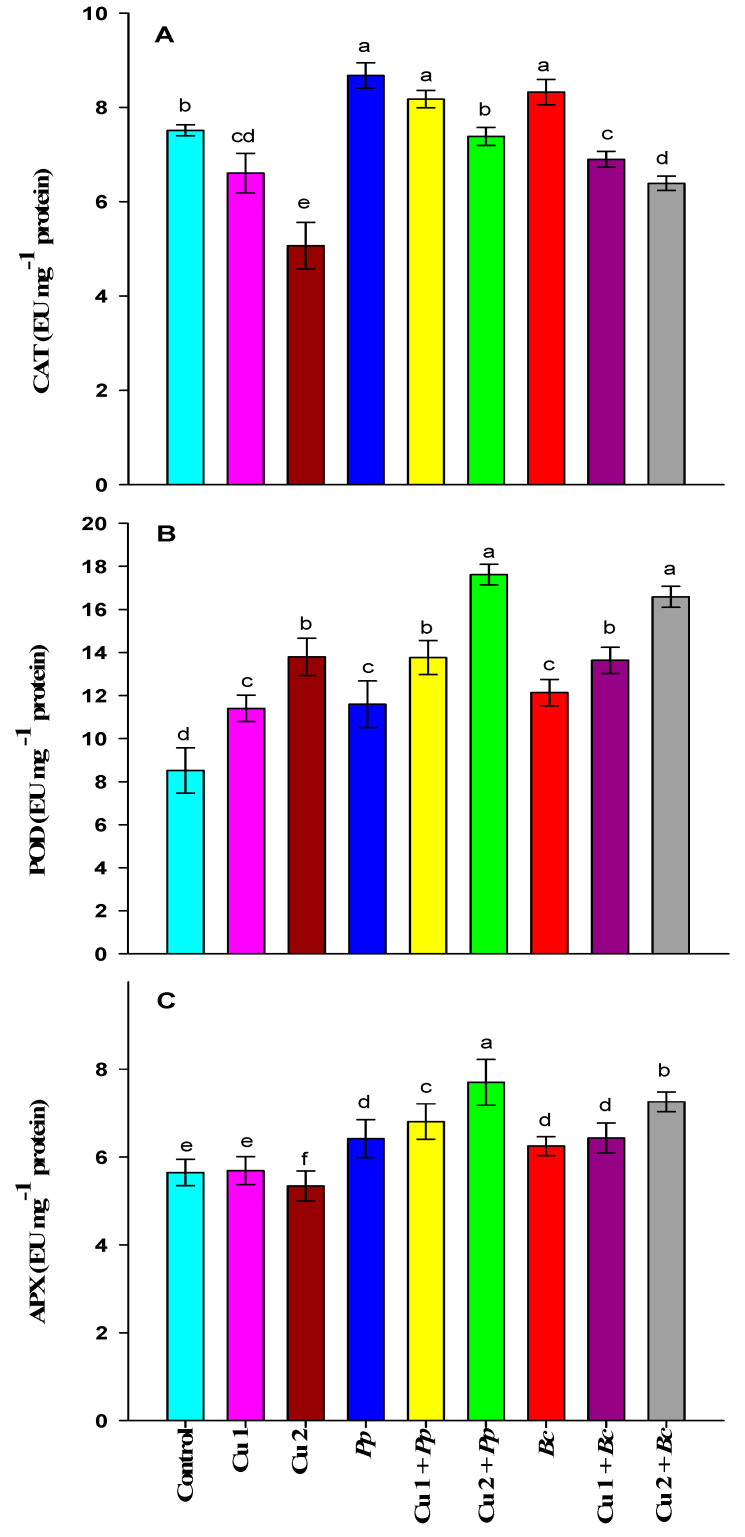
Effects of bacterial inoculation under copper stress on the activities (**A**) catalase (CAT), (**B**) peroxidase (POD), and (**C**) ascorbate peroxidase (APX) in leaves of maize plants. Bars represent the standard deviation (± SD) of the means (*n* = 3). Different letters indicate significant differences among the treatments at *p* ˂ 0.05, according to Duncan’s multiple range test. The treatments include Control, plants without copper stress and bacterial strains inoculation; Cu 1: plants exposed to 100 µM CuSO_4_; Cu 2: plants exposed to 500 µM CuSO_4_; *P. polymyxa* (*Pp*) inoculated plants without CuSO_4_; Cu 1 + *Pp*: plants exposed to 100 µM CuSO_4_ and inoculated with *P. polymyxa*; Cu 2 + *Pp*: plants exposed to 500 µM CuSO_4_ and inoculated with *P. polymyxa*; *B. circulans* (*Bc*) inoculated plants without CuSO_4_; Cu 1 + *Bc*: plants exposed to 100 µM CuSO_4_ and inoculated with *B. circulans* and Cu 2 + *Bc*: plants exposed to 500 µM CuSO_4_ and inoculated with *B. circulans*.

The Cu 1 and Cu 2 doses decreased the activity of CAT significantly compared to controls. The *Pp* and *Bc* inoculation resulted in a marked increment in the activity of CAT compared to control plants. In Cu 1 + *Pp* and Cu 2 + *Pp* plants, the CAT activity was increased over Cu 1 and Cu 2 alone plants. Additionally, *Bc* inoculation in Cu 1 and 2 plants had an additive and significant effect on CAT activity in comparison to plants treated with Cu 1 and 2 alone (Figure 2A).

Maize plants grown with Cu 1 and Cu 2 showed a significant increase in POD activity compared to control plants. A significant increase was also noticed in POD activity when plants were inoculated with *Pp* and *Bc* versus the control plants. A significant increase in the POD activity by *Pp* inoculation under Cu 1 and Cu 2 stress conditions was noticed compared to Cu 1 and Cu 2 alone plants. The POD activity was also incremented significantly with *Bc* inoculation in presence of Cu 1 and Cu 2 compared to Cu 1 and Cu 2 alone plants (Figure 2B).

The Cu 1 dose caused a non-significant in the activity of APX but Cu 2 dose induced a significant increase with respect to control plants. The inoculation with *Pp* and *Bc* in stress-free plants increased APX activity significantly compared to control plants. Treatment of maize plants with Cu 1 and 2 and receiving either *Bc* and/or *Pp* inoculation registered a significant increase in APX activity compared to Cu 1 and 2 alone treated plants (Figure 2C).

### 3.8. Understanding Interactions between Bacterial Inoculation, Various Cu Doses and Variables Studied through PCA Approach

**Figure 3:**
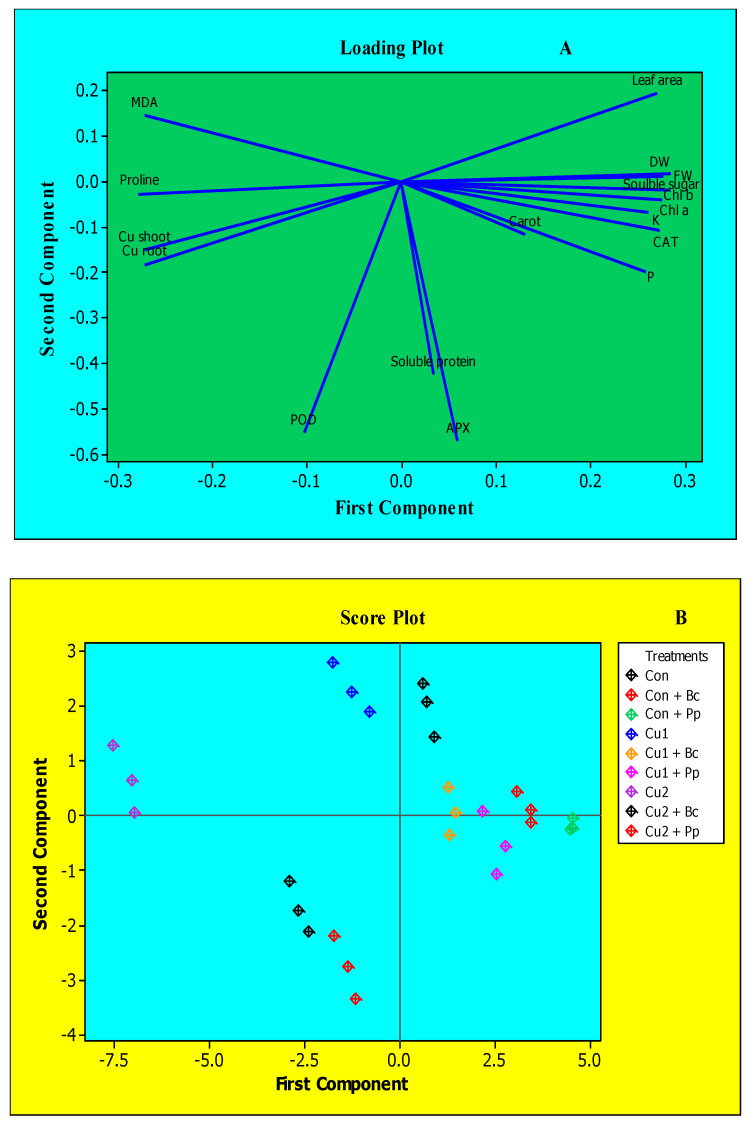
Principal component analysis (PCA) to understand parameter and treatment variability in maize plants. (**A**) The parameters included FW (fresh weight) DW (dry weight), leaf area, Chl *a* (chlorophyll *a*), Chl *b* (chlorophyll *b*), Carot (carotenoids), soluble sugars, soluble protein, proline, MDA (malondialdehyde), CAT (catalase), APX (ascorbate peroxidase), POD (peroxidase), P (phosphorous) and K (potassium). (**B**) The treatment triangles in different colors include Control, plants without copper stress and bacterial strains inoculation; Cu 1: plants exposed to 100 µM CuSO_4_; Cu 2: plants exposed to 500 µM CuSO_4_; *P. polymyxa* (*Pp*) inoculated plants without CuSO_4_; Cu 1 + *Pp*: plants exposed to 100 µM CuSO_4_ and inoculated with *P. polymyxa*; Cu 2 + *Pp*: plants exposed to 500 µM CuSO_4_ and inoculated with *P. polymyxa*; *B. circulans* (*Bc*) inoculated plants without CuSO_4_; Cu 1 + *Bc*: plants exposed to 100 µM CuSO_4_ and inoculated with *B. circulans* and Cu 2 + *Bc*: plants exposed to 500 µM CuSO_4_ and inoculated with *B. circulans*.

A PCA (loading and score plot) was constructed to study the interaction between various variables and doses of Cu to assess the maximum variability of data and to bacterial inoculation treatments. The loading plot shown in Figure 3A of various variables advocated that growth, photosynthetic pigments, organic solutes and antioxidants enzymes were positively correlated with each other and negatively with MDA, proline and Cu root and shoot contents. The score plot (Figure 3B) represented the authenticated grouping of various treatments (bacterial inoculation and Cu doses). The control treatment along with *Bc* and *Pp* inoculation treatments was adjudged as the best value giving treatments. Its effect was followed by that of Cu 1+ *Bc* and Cu 1+ *Pp*. This shows the alleviating effect of *Pp* and *Bc* inoculation in the presence of Cu doses. The Cu doses in the absence of bacterial inoculation impose severe negative growth restrictions in maize plants as both these treatments were clustered on the upper left-hand side of the score plot. The highest dose of Cu (Cu 2) along with *Pp* or *Bc* inoculation were grouped together in the lower two rectangles of the score plot. There was less alleviation in the presence of the highest dose of Cu with bacterial inoculation and this was confirmed by score plot as these treatments were grouped together in a negative component (Figure 3B).

## 4. Discussion

In the present communication, an appraisal has been made to unravel various physio-biochemical mechanisms induced by PGPRs under Cu stress in maize plants. It is well established that the Cu doses induced growth inhibition reflected in the form of fresh and dry weight and leaf area of maize plants (Table 1). It may be attributed to excess Cu phytotoxicity and interruptions in mineral nutrients which resulted in the inhibition of cell division and cell elongation [61], respiration and photosynthesis in plants [62]. Additionally, the reduction in growth traits under Cu-treated maize plants in the present study may also be ascribed to the higher acquisition of excess Cu ions resulting in the reduction of the mineral nutrient contents (Figure 1C,D) which resulted in an inhibition of the functioning of important Cu-containing metalloproteins required to achieve optimum cell growth and metabolism [63]. In contrast, both PGPRs tested to alleviate the Cu phytotoxicity proved effective and increased the aforementioned growth characteristics. The increment in PGPR mediated growth traits could be due to the secretion of various plant hormones in the rhizosphere as well as a tight regulation in the endogenous levels of these plant hormones [64]. These plant hormones are known to maintain cell division and cell elongation processes to a great extent [65], thereby increasing fresh and dry weight as well as leaf area after inoculation with *P. polymyxa* and *B. circulans* in this study. A similar improvement in growth traits by *Bacillus* strains under Mn in *Broussonetia papyrifera* conforms to our present results [66]. A similar increase in growth traits through PGPR inoculation was also recorded in maize plants [67,68,69,70,71].

In the present report, the application of Cu especially Cu 2 also decreased Chl *a*, *b* and Caro contents (Table 2) and this decrement is due to the reaction of Cu with thiol groups of the enzymes like δ-aminolevulinic dehydrogenase and protochlorophyllide reductase complex [72]. Cu induced decreased photosynthetic pigments could also be attributed to impairment in chloroplast structure and composition of thylakoid membrane, photophosphorylation of PS I and PS II, increased photoinhibition and reduction in the photosynthetic electron transport chain [73,74]. A similar decrease in these pigments has been reported in jute [75,76], cilantro [77], lentil [78], spearmint [79] and maize [41] plants. The results of this study showed that in each treatment group of inoculation with *P. polymyxa* and *B. Circulans* increased the photosynthetic pigments under optimal and Cu stress conditions (Table 1). It indicated that the two tested PGPR improved the absorption and utilization of plant minerals and which in turn promoted the biosynthesis of proteins and chlorophyll. The two strains accelerated the absorption and transport of minerals via carriers under Cu stress, which in turn could maintain the synthesis of pigments [66,80,81] even under Cu stress. These results are in harmony with the results of Kamran et al. [82] and Awan et al. [36].

In this study, Cu doses along with PGPR inoculation showed the highest content of osmolytes (soluble sugars and soluble proteins) in the maize plants (Table 2). Increased osmolytes content under Cu stress conditions has aided plants to maintain optimal water balance by regulating turgor and osmotic pressure [10,83,84]. In the present work, elevated content of osmolytes under Cu doses under PGPR inoculation could have preserved sustained redox homeostasis which resulted in optimal plant metabolism. A similar increase in osmolytes content under metal stress by PGPR inoculation was reported in maize [36,85], sunflower [26], rape seedlings [86], potato [24], tomato [87], curtis [88] and lentil [89] plants. Interestingly, the inoculation with *Pp* and *Bc* diminished the accumulation of proline content in the Cu stressed seedlings (Figure 1A,B), which is linked with enhanced growth of maize plants under Cu stress (Table 1). This finding proposed that the inoculation with *Pp* and *Bc* could enable safeguarding of cells by maintaining the accumulation of proline to an optimal level, and PGPR inoculation possibly utilized other osmolytes for Cu stress alleviation, in which the high level of proline accumulation was not needed.

Plants are equipped with antioxidant defense enzyme activities which include SOD, CAT, POD and APX to manage the metal-induced oxidative damage and lessen the accumulation of various ROS. In the present work, the increase in root and shoot Cu content (Figure 1A,B) and MDA level (Table 2) indicates the dose-dependent extent of Cu-induced oxidative damage which negatively affects the membrane’s integrity. In contrast, the antioxidant enzymes (CAT, POD and APX) play the dominant role in alleviating Cu-induced oxidative damage. The activities of antioxidant enzymes, such as CAT, POD, and APX (Figure 2) were increased in maize plants inoculated with PGPR. Notably, the detrimental impact of Cu stress was reduced by the inoculation of PGPR, which significantly upsurges these activities. On one hand, it was observed that PGPR increased antioxidant activities and, on the other decreased root and shoot Cu (Figure 1A,B) and MDA (Table 2) content in maize plants grown under Cu conditions. Khanna et al. [90] stated that PGPR application lowered metal uptake by reducing the expression of metal transporter genes in tomato plants. The present investigation showed that PGPR boosted maize tolerance to Cu stress by minimizing Cu accumulation and translocation from root to shoot, thus protecting maize against Cu toxicity and enhancing maize growth. Our findings are also supported by Fatnassi et al. [91], who stated that the increased antioxidant enzymes activity inoculated with the PGPRs reduced Cu stress in *Vicia faba* plants. In yet another study, Ju et al. [92] reported the impact of co-inoculation of PGPR and rhizobium on the biochemical responses of alfalfa plants under Cu stress conditions and found that inoculating plants decreased MDA content on one hand and increased antioxidant activities on the other, thus further supporting our present findings.

Our results determined that the application of Cu to maize plants decreased P and K levels (Figure 1C,D). Cu is known to reduce the uptake and aggregation of plant nutrients due to direct competition with these nutrients [93]. A similar Cu mediated decrease in mineral nutrients has been reported in maize [42,94,95], which is in line with our current findings. On the other hand, we observed that *P. polymyxa* and *B. circulans* inoculation significantly increased these contents under Cu doses. It has been reported that *P. polymyxa* stimulates the plant’s nutrient acquisition machinery by transcriptionally activating the nutrient-deficiency-induced transcription factors in addition to secreting auxins and cytokinins [96,97,98]. The increased availability of nutrient uptake by *P. polymyxa* inoculation in the presence of Cu doses could have increased nutrient transport in maize plants which resulted in an increase in P and K contents. Nevertheless, *Bacillus* strains are also known to solubilize soil P pools, thus increasing its uptake and translocation to plants [99]. A similar increase in plant mineral nutrients by the inoculation of *Bacillus* strains has been recorded in maize plants [69,100,101], which are in support of our present findings.

PCA has further confirmed a strong correlation between the inoculation of PGPR strains and plant physio–biochemical attributes under Cu stress conditions. For the determination of approximate association of various biochemical and morpho-physiological traits and different treatments, loading and score plots were constructed (Figure 3A,B). PCA results validated the cluster analysis of different treatments. For example, Control (Con), Con + *Bc* and Con + *Pp* treatments were grouped together, and these three treatments were associated with LA, chlorophyll *a* and *b*, FW, DW, soluble sugar. This means that these two strains of bacteria treatment increased these traits in a significant manner. The low dose of Cu treatments along with PGPR inoculation like Cu 1 + *Bc* and Cu 1 + *Pp* were also grouped together. These two treatments represented the maximum alleviation effect under low Cu dose. The two Cu (Cu 1 and Cu 2) doses without PGPR inoculation were found to be grouped together. These treatments represented the toxic effect of Cu doses on various biochemical and morpho-physiological traits on maize plants. The treatments Cu 2 + *Bc* and Cu 2 + *Pp* depicted the minimum alleviation effect by PGPR inoculation under high Cu doses and were both grouped together.

## 5. Conclusions

The present work on maize plants showed that Cu doses increased root and shoot Cu content, proline, MDA content, POD activity and decreased most of the biochemical and morpho-physiological characters in a dose-dependent manner. The inoculation with PGPR strains has shown a significant decrease in stress caused by the Cu doses on all studied plant attributes by improving pigments and mineral nutrients, reducing the accumulation of Cu in root and shoot, and augmentation the ROS scavenging system via an elevated antioxidant defense system. The antioxidant defense system and osmolytes play a central role in the alleviation of Cu toxicity of maize plants which in turn improved maize growth.

## Figures and Tables

**Table 1:** Effects of bacterial inoculation under copper stress on fresh weight (g plant^−1^) (b) dry weight (g plant^−1^) and (c) leaf area (cm^2^ plant^−1^) in maize plants. Bars represent the standard deviation (±SD) of the means (*n* = 3). Different letters indicate significant differences among the treatments at *p* ˂ 0.05, according to Duncan’s multiple range test. The treatments include Control, plants without copper stress and bacterial strains inoculation; Cu 1: plants exposed to 100 µM CuSO_4_; Cu 2: plants exposed to 500 µM CuSO_4_; *P. polymyxa* (*Pp*) inoculated plants without CuSO_4_; Cu 1 + *Pp*: plants exposed to 100 µM CuSO_4_ and inoculated with *P. polymyxa*; Cu 2 + *Pp*: plants exposed to 500 µM CuSO_4_ and inoculated with *P. polymyxa*; *B. circulans* (*Bc*) inoculated plants without CuSO_4_; Cu 1 + *Bc*: plants exposed to 100 µM CuSO_4_ and inoculated with *B. circulans* and Cu 2 + *Bc*: plants exposed to 500 µM CuSO_4_ and inoculated with *B. circulans*.

Treatments	Fresh Weight	Dry Weight	Leaf Area
**Control**	5.39 ± 0.27 ^c^	0.46 ± 0.02 ^c^	23.06 ± 0.14 ^e^
**Cu 1**	4.77 ± 0.10 ^d^	0.40 ± 0.02 ^d^	22.33 ± 0.11 ^f^
**Cu 2**	3.28 ± 0.05 ^f^	0.26 ± 0.03 ^f^	8.71 ± 0.14 ^i^
***Pp***	6.70 ± 0.15 ^a^	0.61 ± 0.01 ^a^	27.51 ± 0.06 ^a^
**Cu 1 + *Pp***	5.26 ± 0.14 ^c^	0.47 ± 0.02 ^c^	26.61 ± 0.08 ^b^
**Cu 2 + *Pp***	4.87 ± 0.09 ^d^	0.41 ± 0.02 ^d^	14.48 ± 0.14 ^g^
***Bc***	6.20 ± 0.11 ^b^	0.54 ± 0.02 ^b^	25.24 ± 0.08 ^c^
**Cu 1 + *Bc***	5.15 ± 0.12 ^c^	0.43 ± 0.02 ^cd^	24.37 ± 0.08 ^d^
**Cu 2 + *Bc***	4.40 ± 0.13 ^e^	0.36 ± 0.02 ^e^	12.53 ± 0.22 ^h^

**Table 2:** Effects of bacterial inoculation under copper stress on chlorophyll a (Chl *a*), chlorophyll b (Chl *b*), carotenoids (Caro), soluble sugars, soluble proteins, proline (mg g^−1^ fresh weight (FW)), and malondialdehyde (MDA; nmol g^−1^ FW) contents in maize plants. Bars represent the standard deviation (± SD) of the means (*n* = 3). Different letters indicate significant differences among the treatments at *p* ˂ 0.05, according to Duncan’s multiple range test. The treatments include Control, plants without copper stress and bacterial strains inoculation; Cu 1: plants exposed to 100 µM CuSO_4_; Cu 2: plants exposed to 500 µM CuSO_4_; *P. polymyxa* (*Pp*) inoculated plants without CuSO_4_; Cu 1 + *Pp*: plants exposed to 100 µM CuSO_4_ and inoculated with *P. polymyxa*; Cu 2 + *Pp*: plants exposed to 500 µM CuSO_4_ and inoculated with *P. polymyxa*; *B. circulans* (*Bc*) inoculated plants without CuSO_4_; Cu 1 + *Bc*: plants exposed to 100 µM CuSO_4_ and inoculated with *B. circulans* and Cu 2 + *Bc*: plants exposed to 500 µM CuSO_4_ and inoculated with *B. circulans*.

Treatments	Chl *a*	Chl *b*	Caro	Soluble Sugars	Soluble Proteins	Proline	MDA
**Control**	0.34 ± 0.03 ^c^	0.25 ± 0.03 ^c^	0.14 ± 0.02 ^bc^	122.49 ± 3.68 ^bc^	124.92 ± 3.49 ^ab^	3.54 ± 0.14 ^e^	41.48 ± 1.00 ^cd^
**Cu 1**	0.26 ± 0.03 ^d^	0.24 ± 0.03 ^c^	0.18 ± 0.02 ^abc^	113.52 ± 3.08 ^de^	97.96 ± 2.78 ^c^	5.86 ± 0.13 ^b^	46.94 ± 1.70 ^b^
**Cu 2**	0.09 ± 0.02 ^f^	0.13 ± 0.01 ^e^	0.09 ± 0.08 ^c^	84.30 ± 3.77 ^g^	126.24 ± 6.74 ^ab^	7.19 ± 0.14 ^a^	65.22 ± 0.91 ^a^
***Pp***	0.50 ± 0.02 ^a^	0.43 ± 0.02 ^a^	0.15 ± 0.09 ^abc^	137.78 ± 2.27 ^a^	133.41 ± 3.30 ^a^	2.77 ± 0.06 ^g^	30.75 ± 1.66 ^g^
**Cu 1 + *Pp***	0.42 ± 0.03 ^b^	0.37 ± 0.03 ^b^	0.23 ± 0.06 ^a^	126.42 ± 3.12 ^b^	120.01 ± 9.55 ^b^	3.70 ± 0.08 ^de^	32.76 ± 1.00 ^fg^
**Cu 2 + *Pp***	0.27 ± 0.01 ^de^	0.23 ± 0.02 ^cd^	0.19 ± 0.03 ^ab^	108.87 ± 4.14 ^ef^	135.18 ± 5.87 ^a^	5.26 ± 0.10 ^c^	39.90 ± 2.44 ^d^
***Bc***	0.49 ± 0.03 ^a^	0.37 ± 0.02 ^b^	0.18 ± 0.03 ^abc^	132.89 ± 3.83 ^a^	129.25 ± 3.49 ^ab^	3.06 ± 0.08 ^f^	33.35 ± 1.14 ^ef^
**Cu 1 + *Bc***	0.43 ± 0.02 ^b^	0.33 ± 0.02 ^b^	0.22 ± 0.05 ^ab^	118.14 ± 2.45 ^cd^	118.83 ± 7.83 ^b^	3.86 ± 0.08 ^d^	35.64 ± 0.51 ^e^
**Cu 2 + *Bc***	0.22 ± 0.03 ^e^	0.19 ± 0.02 ^d^	0.14 ± 0.03 ^abc^	105.86 ± 6.03 ^f^	127.62 ± 6.57 ^ab^	5.65 ± 0.22 ^b^	43.12 ± 1.11 ^c^
